# Face, head, and neck (FHN) problems and surgical need assessment among refugees living in Tanzania

**DOI:** 10.1371/journal.pgph.0004170

**Published:** 2026-01-30

**Authors:** Ashwin Ram Reddy, Alexander J. Blum, Hilary Ngude, Frank Manyama, Omar Juma, Mohamed Yunus Rafiq, Joseph V. Sakran, Kent Stevens, Zachary Obinna Enumah

**Affiliations:** 1 Johns Hopkins University School of Medicine, Baltimore, Maryland, United States of America; 2 Tanzania Red Cross Society, Dar es Salaam, Tanzania; 3 Ifakara Health Institute, Ifakara, Morogoro, Tanzania; 4 NYU Shanghai, Shanghai, China; 5 Center for Global Surgery, Department of Surgery, Johns Hopkins Hospital, Baltimore, Maryland, United States of America; Augusta University, UNITED STATES OF AMERICA

## Abstract

Otolaryngology-head and neck surgery (OHNS) conditions comprise a considerable and underprioritized portion of the global burden of surgical disease. While OHNS conditions have been found to disproportionately impact low- and middle-income countries (LMICs), no studies to date have sought to characterize the burden of OHNS disease within chronic refugee settings in LMICs. The cluster-randomized, cross-sectional Surgeons Overseas Assessment of Surgical Need (SOSAS) survey tool was administered in Nyarugusu Refugee Camp in Makere, Tanzania in August and September of 2021 to estimate the prevalence of face, head, and neck (FHN) disease among refugees in East Africa and to identify predictors of surgical need. Demographic and clinical data were collected from all study participants. A multivariable logistical model was used to identify demographic and clinical characteristics associated with increased surgical need. 462 refugees reported having an FHN condition within their lifetime and 242 (52%) indicated a current surgical need for an FHN problem. Most conditions (n = 335; 73%) had persisted for over a year. The most common pathologies were non-injury-related wounds (n = 146; 35%) and non-congenitally acquired malformations (n = 118;28%). Multivariable analysis revealed several factors associated with increased surgical need including age, nationality, religion, and pathology type. There is a high burden of untreated surgical FHN conditions in Nyarugusu Refugee Camp. This study underscores the need for targeted interventions to expand otolaryngology-head and neck surgery (OHNS) services for FHN problems in under-resourced, protracted refugee settings. In addition to investing in OHNS surgical training programs, we advocate for temporizing interim non-surgical management while pursuing expedited referral for definitive surgical intervention.

## Introduction

The delivery of high-quality surgical care is a critical component in a comprehensive healthcare system, yet approximately five billion globally lack access to safe, timely, and affordable surgical care [1]. A considerable burden of surgical disease is attributable to otolaryngology–head and neck surgery (OHNS) conditions such as hearing loss, head and neck cancer, and craniofacial trauma [2]. Without appropriate intervention, head and neck conditions such as tumors, infections, and trauma-related deformities can disrupt key functions like breathing and eating, leading to increased risks respiratory complications, malnutrition, and mortality. Additionally, conditions such as visible facial deformities and hearing loss can foster stigma and isolation, posing mental health challenges and curtailing opportunities for social and economic integration. In under-resourced environments such as refugee settings in low- and middle-income countries (LMICs), this places additional strain on family support networks and limited healthcare resources. Despite the known disproportionate impact of OHNS disease in LMICs [2–4], research specifically addressing the burden of these conditions within refugee settings remains limited.

There are over 36 million refugees worldwide, 75% of whom are hosted in LMICs [5]. Refugees often present to care in settings with limited resources and constrained health systems; they are thus particularly predisposed to lacking access to safe, timely, and affordable surgical care. A 2014 analysis estimated that 3 million additional surgical procedures are needed for all displaced persons worldwide, although this number is likely an outdated underestimate as there are twice as many displaced persons today [6]. While prior studies have sought to characterize the types of OHNS disease in refugees who have migrated to high-income countries [7–8], there are no identifiable published studies assessing the burden of OHNS disease in protracted refugee camp settings in sub-Saharan Africa. Our group previously described the burden of untreated surgical conditions among refugees in Nyarugusu Refugee Camp using the validated Surgeons Overseas Assessment of Surgical Need (SOSAS) tool [9]. Stemming from that parent study, this work seeks to more closely characterize the burden of untreated OHNS surgical problems and to explore factors associated with surgical need for face, head, or neck (FHN) problems among refugees in Nyarugusu Refugee Camp.

## Materials and methods

### Study setting

This study was conducted in Nyarugusu Refugee Camp, located in the Kigoma region of Western Tanzania, from August 4 to September 10, 2021. The camp was established in 1996 and is one of three main refugee camps in Tanzania. Over 130,000 refugees from the Democratic Republic of Congo (DRC) and Burundi currently reside in the camp. Despite intermittent repatriation of some refugees back to Burundi, the majority of Nyarugusu’s inhabitants are currently unable to return to their homeland and have not been granted permanent residence in another country. Nyarugusu Refugee Camp, having hosted refugees for more than five consecutive years, is considered a protracted refugee setting [10].

### Data collection

Data for this study is drawn from our parent study that assessed the burden of surgical disease among 3,574 refugees living in Nyarugusu Refugee Camp using the previously validated Surgeons OverSeas Assessment of Surgical Need (SOSAS) tool [9,11]. To date, the SOSAS tool has been utilized to estimate the prevalence of surgical disease in several LMICs and in one other refugee population [12]. It involves conducting a verbal head-to-toe examination to identify current or prior problems that may have required (or may be amenable to) surgical management. The survey is divided into the subsequent anatomical regions: face/head/neck (including the eye), extremities, abdomen, back, chest/breast, and groin. Derived from a sampling framework based on the administrative divisions of zones, villages, clusters, and households in Nyarugusu, 126 clusters were randomly selected for administration of the SOSAS tool. Every household in a cluster was approached, and two individuals from each household were chosen at random to participate. There was a 99% response rate. Fourteen refugee health educators carried out data collection after an extensive training process. A local research manager supervised the data collectors. Data was collected using an Android tablet equipped REDCap Mobile with offline functionality. Data was uploaded to the Johns Hopkins REDCap database on a daily basis. Only individuals endorsing a face, head, and neck problem unrelated to the eye were included in this analysis.

### Data analysis

Data analysis was conducted using Stata 16 (Stata 16, College Station, TX). For descriptive analysis, categorical variables were presented as a total number with a relevant percentage and continuous variables were reported as a mean with a standard deviation or a median with an interquartile range. Additionally, we used a multivariable regression model to explore predictors of presence of a current FHN surgical problem. To identify predictors of having a current FHN surgical problem, we specified a multivariable logistic regression model. Covariates were selected *a priori* based on their theoretical and clinical relevance to surgical need in refugee populations. Specifically, we included age, sex, nationality, religion, time course of symptoms, anatomical region, and pathology type because these factors represent key demographic or clinical characteristics that could confound the relationship between presenting complaint and perceived surgical need or influence a refugee’s ability to access care within a strained health system. Reference groups included age under 18, male sex, Burundian nationality, Christian religion, time course of over twelve months or more, anatomical location of head, and pathology type of injury-related wound. Reference groups for variables including Christian religion, time course, and anatomical location were chosen based on the highest overall frequency in the sample population. Injury-related wounds were selected as the reference group for pathology type as they represent a universally recognized, acute condition that is typically prioritized for treatment, serving as a baseline to compare access disparities with other elective FHN conditions. Multivariable analysis findings were presented as odds ratios with 95% confidence intervals. Cluster-correlated robust standard errors were used to calculate 95% confidence intervals to account for potential within-cluster non-independence of outcomes.

### Ethical approval

Approval for this study protocol, including the consent process, was granted by the Johns Hopkins Medicine Institutional Review Board (IRB00258009) and the Tanzanian Commission on Science and Technology (2020–391-NA-2011–143). The Tanzanian Ministry of Home Affairs provided authorization to enter the camp. Informed consent was verbally obtained from all adult participants directly. Parents or other adult household members verbally consented on behalf of children under the age of 18. The consent process was conducted by trained health educators, who underwent standardized training on obtaining informed consent to ensure comprehension and ethical compliance. If consent was not obtained, the participant was not enrolled in the study. This information was recorded as a variable within the study dataset to ensure accountability. Participants were informed that their involvement was voluntary, would not affect their eligibility for or quality of treatment, and that they could withdraw from the study at any time.

## Results

### Demographics

462 refugees endorsed having a potentially surgically correctable pathology in the face, head, and neck (FHN). The average age was 28.3 (SD 18.0) with the most represented group being pediatric patients under the age of 18 (n = 142; 31%). More than half of those endorsing FHN problems were women (n = 285; 62%). Over two thirds were originally from the DRC (n = 312; 68%), while the rest were from Burundi. There was an even distribution across educational backgrounds (no education, primary school education, and secondary school education), with each group accounting for about a third of all refugees with a reported FHN condition. Only 6 individuals (1%) had attained higher education. Most of those with a FHN condition were unemployed (n = 244; 53%). The vast majority identified as Christian (n = 419; 91%). Over half were literate (n = 271; 59%). Table 1.

**Table 1:** Demographics.

	Total
N	462
Average age (SD)	28.3 (18.0)
Age categories	
Under 18 Years	142 (30.8%)
Age 18–29	126 (27.3%)
Age 30–44	104 (22.6%)
Age 45–59	48 (10.4%)
Age 60 or Older	41 (8.9%)
Sex	
Male	177 (38.3%)
Female	285 (61.7%)
Country of origin	
Democratic Republic of the Congo	312 (67.5%)
Burundi	150 (32.5%)
Education	
None	152 (32.9%)
Primary school	149 (32.3%)
Secondary school	155 (33.5%)
Higher education	6 (1.3%)
Occupation	
Unemployed	244 (52.8%)
Farmer	79 (17.1%)
Small business owner	27 (5.8%)
Home helper	2 (0.4%)
Self employed	23 (5.0%)
Housewife	33 (7.1%)
Other	54 (11.7%)
Religion	
Christian	419 (91.3%)
Muslim	28 (6.1%)
Other	12 (2.6%)
Literacy	
No	190 (41.2%)
Yes	271 (58.8%)

### Baseline health characteristics

Almost three quarters of refugees endorsing a FHN problem considered themselves to be in good health (n = 325; 71%). Almost all had previously sought medical care at a primary health center in Nyarugusu (n = 447; 97%). A majority noted some kind of illness within the past year (n = 373; 81%) with a median of three weeks of illness (IQR 1–5) and three visits to a health center (IQR 2–5). Almost two thirds reported having recovered from their illness (n = 244; 65%). Table 2.

**Table 2:** Baseline health characteristics.

	Total
N	462
Median amount of time ill in weeks (IQR)	3 (1-5)
Median number of visits to health center (IQR)	3 (2-5)
Are you in good health? - Yes	325 (70.7%)
Are you in good health? - No	135 (29.3%)
Have you sought medical care in Nyarugusu? - Yes	447 (97.0%)
Have you sought medical care in Nyarugusu? - No	14 (3.0%)
Have you been ill at all in the past year? - Yes	373 (80.9%)
Have you been ill at all in the past year? - No	88 (19.1%)
Have you recovered from illness? - Yes	244 (65.4%)
Have you recovered from illness? - No	129 (34.6%)

### Characteristics of FHN disease in Nyarugusu Refugee Camp

**Fig 1:**
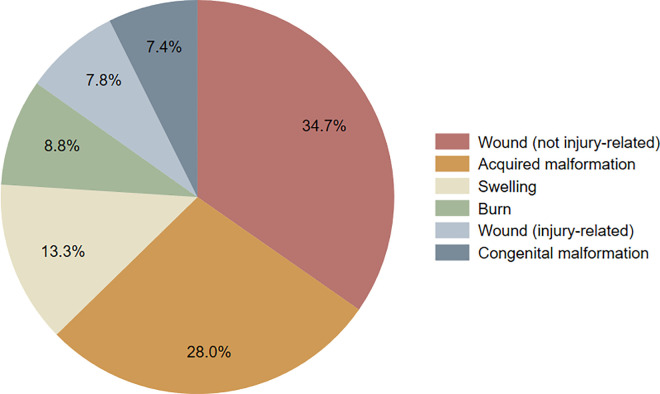
Breakdown of FHN problem pathologies. Pie chart displaying the breakdown of reported FHN problems by pathology. Pathology categories are listed from highest to lowest proportion. The percentage associated with each reported pathology is also shown.

Most participants reporting FHN disease characterized it as chronic (i.e., experienced for over a year; n = 335; 73%). Almost half of all endorsed FHN disease was related to the anatomical region of the head (n = 209; 45%), while fewer participants reported problems related to the ear/nose/throat (n = 98 21%), neck (n = 97; 21%), and teeth/mouth (n = 58; 13%). FHN problems were most commonly caused by non-injury-related wounds (n = 146; 35%) and acquired malformations (n = 118; 28%). They were least commonly caused by congenital malformations (n = 31; 7.4%) and injury-related wounds (n = 33; 7.8%) (Fig 1). Over half of respondents noted that their problem was currently affecting them (n = 242; 53%) and almost three quarters reported that they had sought healthcare for their problem (n = 332; 72%). Up to an eighth had seen a traditional healer for their problem (n = 60; 13%). More than half characterized their disease as debilitating (n = 262; 57%). Table 3.

**Table 3:** Characteristics of FHN disease in Nyarugusu Refugee Camp.

	Total
N	462
Time course	
Last month	60 (13.0%)
Between 1–12 months ago	66 (14.3%)
Over 12 months ago	335 (72.7%)
Location of problem	
Ear/nose/throat	98 (21.2%)
Neck	97 (21.0%)
Teeth/mouth	58 (12.6%)
Head	209 (45.2%)
Details of problem	
Acquired malformation	118 (28.0%)
Burn	37 (8.8%)
Congenital malformation	31 (7.4%)
Swelling (mass or growth, goiter)	56 (13.3%)
Wound (injury-related)	33 (7.8%)
Wound (not injury-related)	146 (34.7%)
Is the problem currently affecting you?	
No	217 (47.3%)
Yes	242 (52.7%)
Did you seek healthcare?	
No	130 (28.1%)
Yes	332 (71.9%)
Did you see a traditional healer?	
No	400 (87.0%)
Yes	60 (13.0%)
Is the problem debilitating?	
No	199 (43.2%)
Yes	262 (56.8%)

### Top reasons for not having surgery

A subset of the total respondents (n = 278; 60%) shared why they did not receive surgical care for their FHN problems at the time of being interviewed. Over half (n = 149; 58%) of these respondents reported that they did not perceive a need for surgery. The other two most cited reasons were scarcity of appropriate services (n = 65; 25%) and no money for treatment (n = 25; 8%). Table 4.

**Table 4:** Top Reasons for not having surgery for FHN problem.

	Total
N	256*
Why did you not get surgery for your FHN problem?	
Did not know about access to treatment	3 (1.2%)
Fear/mistrust	8 (3.1%)
Lack of services (facilities/staff/equipment)	65 (25.4%)
No money for travel	2 (0.8%)
No money for treatment	20 (7.8%)
No perceived need	149 (58.2%)
No time	9 (3.5%)

*22 (7.9%) of the original 278 removed from table as they indicated they did receive surgery

### Predictors of current surgical need

The results of the multivariable regression model are displayed in Table 5. Participants aged 45–59 were 2.69 times (95% CI 1.06 - 6.79) more likely to report an active surgical FHN problem compared to the reference group of age under 18. Congolese individuals were 2.15 times (95% CI 1.19 - 3.88) more likely to report an active surgical FHN problem compared to Burundian individuals. Participants who identified as Muslim were 83% less likely (OR=0.17, 95% CI 0.07 - 0.41) to report an active surgical FHN problem compared to those identifying as Christian. Relative to other anatomically defined head-region problems, ear/nose/throat pathologies demonstrated higher odds of reported surgical need (OR = 1.97; 95% CI 1.15–3.34).When referenced against injury-related wounds, congenital malformations (OR=13.69; 95% CI 3.38 - 55.51), acquired malformations (OR=7.83; 95% CI 2.48 - 24.66), and non-injury-related wounds (OR=6.27; 95% CI 2.03- 19.32) were all associated with a much higher odds of reported current surgical need. No statistically significant odds differences were noted by sex or time course.

**Table 5:** Multivariable regression model.

Covariates	Odds Ratio	P-value	95% Confidence Interval
Age categories			
Under 18	REF	–	–
Age 18–29	1.24	0.389	0.75 - 2.06
Age 30–44	1.43	0.240	0.79 - 2.61
Age 45–59	2.69	0.037*	1.06 - 6.79
Age 60 or Older	2.16	0.053	0.99 - 4.72
Sex			
Male	REF	–	–
Female	1.22	0.412	0.76 - 1.97
Country of origin			
Burundi	REF	–	–
Democratic Republic of the Congo	2.15	0.011*	1.19 - 3.88
Religion			
Christian	REF	–	–
Muslim	0.17	0.000***	0.07 - 0.41
Other	1.50	0.643	0.27 - 8.22
Time course of FHN problem			
Over 12 months ago	REF	–	–
Between 1–12 months ago	0.63	0.160	0.34 - 1.20
Less than one month	0.75	0.526	0.31 - 1.83
Location of FHN problem			
Head	REF	–	–
Ear/nose/throat	1.97	0.012*	1.15 - 3.34
Neck	0.85	0.603	0.45 - 1.59
Teeth/Mouth	1.41	0.321	0.71 - 2.79
Details of FHN problem			
Wound (injury-related)	REF	–	–
Acquired malformation	7.83	0.000***	2.48 - 24.66
Burn	1.15	0.867	0.22 - 6.01
Congenital malformation	13.69	0.000***	3.38 - 55.51
Swelling (mass or growth, goiter)	3.26	0.057	0.96 - 11.01
Wound (not injury-related)	6.27	0.001**	2.03- 19.32

*Statistically significant (p < 0.050), ** Highly significant (p < 0.010), *** Very highly significant (p < 0.001)

## Discussion

### Burden of OHNS disease in a protracted/chronic refugee setting

To our knowledge, this study is one of the first to characterize OHNS conditions in a protracted refugee camp setting. Our parent study found that 13% of refugees in Nyarugusu Camp endorsed a lifetime FHN problem (excluding eye pathologies) that may be amenable to surgery [9]. In this study, we found that 53% of these reported FHN problems were unresolved, 57% had debilitating impacts on refugees’ quality of life, and 73% had been ongoing for over a year. The high prevalence of active FHN problems in a chronic refugee setting such as Nyarugusu underscores a significant and overlooked public health burden within the refugee health agenda [13]. In 2023, 99% of households in the camp accessed clean drinking water, only 1.7% of children under five experienced acute malnutrition, and there were no refugee maternal deaths among 6,235 births [14,15]. The presence of unresolved FHN problems among 7% of 3,574 surveyed refugees in Nyarugusu highlights the necessity of ensuring access to OHNS services as a public health priority in chronic refugee settings. Regarding the classification of FHN pathology types, our findings are consistent with those from other studies that utilized the SOSAS tool in non-refugee settings in sub-Saharan Africa [16–18]. While injury-related wounds accounted for 21% and 29% of reported FHN surgical problems in non-refugee studies from Sierra Leone and Uganda, injury-related wounds were the least represented pathology type in Nyarugusu Refugee Camp, accounting for less than 8% of FHN surgical problems. Additionally, when compared to injury-related wounds in multivariable analysis, non-acute pathologies such as congenital malformations, acquired malformations, and non-injury-related wounds were each associated with much higher odds of reporting ongoing need for surgery. Our study adds to existing literature to show that surgical needs in protracted refugee camp settings are predominantly non-trauma-related and more elective in nature [19,20]. These findings stand in contrast to surgical problems commonly encountered in active conflict zones and recently established refugee camps housing individuals fleeing physical violence and persecution [21].

### Building OHNS services in refugee camps

In our study, 31% of refugees with reported ongoing surgical problems indicated that they had not received surgical care due to a lack of services. Building capacity for OHNS care in Nyarugusu Refugee Camp is essential to addressing the high prevalence of chronic, untreated FHN surgical problems reported by study participants. The provision of OHNS services in the camp is primarily limited by lack of personnel and equipment. General practitioners often perform surgical procedures such as caesarean sections due to the absence of formally trained specialists. Additionally, constraints in anesthesia capabilities limit surgical procedures to those that can be performed under spinal anesthesia or without intubation. Tanzania only has 0.05 physician anesthesiologists per 100,000 population and 0.15 anesthesia providers of any type per 100,000 population [22,23]. Thus, many patients with surgical conditions are referred to surrounding district hospitals. This is especially true for refugees with FHN conditions. One study of referrals from Nyarugusu Refugee Camp to Kabanga Hospital found that otolaryngology was the third most frequently referred-to specialty after ophthalmology and general surgery [24]. Navigating the referral process as a refugee is difficult and unpredictable. Financial, logistical, and security constraints severely limit the number of refugees who ultimately receive referrals. Refugees may subsequently encounter referral delays and long wait times that enable their FHN problems to persist until they are chronic in nature. Delaying OHNS problems for referral to secondary care can lead to increased morbidity, heightened risk of complications, and poor patient outcomes. Receiving follow-up care can be similarly challenging for all the same reasons. We advocate for increasing the capacity of OHNS services in refugee camps. Building specialty care in places like Nyarugusu refugee camp takes time and must be done thoughtfully in partnership with host communities and existing organizations such as the Tanzania Red Cross Society that already provides medical care in the camp. As suggested in previous studies, short term efforts may include increasing the number of visiting specialist surgeons and training general surgeons in OHNS procedures [25,26]. Alongside these models, urgent investment in general and subspecialty OHNS training programs in host countries is necessary to create a sustainable workforce in the long term.

### Inclusion of OHNS care in surgical capacity building policies

Our parent study found that 13% of refugees in Nyarugusu Refugee Camp endorsed non-eye-related FHN problems, and this current study found that 53% of these problems remain unresolved. Extrapolating these findings to the 246,000 individuals who comprise the total refugee population in Tanzania [27], we estimate that almost 17,000 OHNS procedures are needed to treat the burden of unresolved FHN problems among all refugees in Tanzania. To build OHNS services for all Tanzanian refugees and the country’s population at large, OHNS care ought to better represented in the country’s national health policies and plans focused on improving surgical capacity and access. The National Surgical, Obstetric, and Anesthesia Plan (NSOAP) is one such example of an established policy framework that addresses the country-specific health burden of conditions requiring surgery. Although Tanzania’s NSOAP for 2018–2025 lays out systematic and comprehensive steps to improve surgical, obstetric, and anesthesia care in the country, the plan does not mention efforts to build capacity for OHNS care [28]. Similarly, there are no mentions of otolaryngology or head and neck surgery in the Tanzanian Health Sector Strategic Plan for 2021–2026 [29]. As described in our parent study, FHN problems were the most common site for potentially surgically correctable pathologies in Nyarugusu Refugee Camp. We believe that any future policies and plans for surgical capacity building must also include OHNS care.

### Limitations

This study has several limitations. Our verbal head-to-toe examination did not include a physical examination—or further workup (i.e., imaging)—by a trained physician or surgeon, as this would have required extensive resources. Nevertheless, previous literature using the SOSAS tool in other contexts suggests a high correlation between verbal head-to-toe examination and visual physical examination [30]. Participants and surveyors in our study did not categorize problems as surgical or non-surgical. Instead, this determination was based on pathology types (e.g., swelling, burn, wound, congenital or acquired disability, history of operation, etc.) that may be amenable to minor or major surgeries. For this reason, we may have included some problems not amenable to surgery, leading to an overestimation of the prevalence of surgical problems. There is also potential for underestimation, as conditions such as hearing loss or asymptomatic masses are unlikely to be reported. Additionally, because surgical status was not systematically collected for all respondents—and only a subset reported reasons for not receiving surgery—we were unable to generate complete comparisons between individuals who did and did not undergo surgery. Because the SOSAS tool uses very generalized categorizations for both anatomical location (i.e., head, neck, teeth/mouth, and ear/nose/throat) and pathology type, we are unable to assess the burden of specific conditions (e.g., chronic otitis media, thyroid malignancy, septal deviation, etc.) and estimate specific workforce and equipment needs. Lastly, unlike previous studies in Nyarugusu Refugee Camp, this study did not include residential zone as a covariate in our final multivariable regression model. While camp zones may serve as a rough proxy for distance from the camp’s main surgical center, this distance can vary greatly even within a single residential zone as each zone encompasses a large geographic area. Additionally, as the camp continues to grow, zone boundaries are constantly redrawn. Thus, any appreciable differences in surgical need between zones would not provide very meaningful information. It is important to note that, when zone is added to the regression model, no significant differences in nationality are noted. Instead, certain zones that are primarily comprised of Congolese inhabitants are associated with higher odds of having surgical need. To better understand how distance from the surgical center contributes to surgical need, further research with geographic information system technology is warranted.

### Strengths

Our study offers a significant advancement in the understanding of the burden of FHN conditions in a chronic refugee setting. A major strength of this study is its 99% response rate, which minimizes non-response bias and enhances the representativeness of our findings, allowing for more reliable conclusions regarding health needs in a refugee camp population. Additionally, by using a cluster-randomized, cross-sectional approach which accounted for the organization of refugee shelters, our study provides insights into disease burden and surgical needs efficiently, bypassing the need for individual data collection across the population—a model particularly feasible for significantly resource-constrained settings such as Nyarugusu Refugee Camp. Similarly, because the SOSAS tool was administered by trained refugee health educators at the household, rather than health facility level, we did not strain the already limited health infrastructure in the camp. Finally, our study highlights key financial and logistical barriers to surgical care, offering valuable insights for policymakers and camp administrators to formulate interventions to enhance surgical access within the camp.

## Conclusion

This is the first study we are aware of that demonstrates the reported prevalence of untreated OHNS disease in a protracted refugee setting in sub-Saharan Africa. We characterize this disease burden by anatomical region, pathology type, and chronicity and identify predictors of increased reported surgical need. Our findings also highlight the lack of OHNS services within refugee camps. There is great need for targeted interventions aimed at expanding the OHNS surgical workforce and operative capacity in chronic refugee settings. As the number of refugees and displaced persons worldwide continues to surge, it is imperative to include this population in global surgery advocacy and capacity building endeavors.
